# QUALITY OF LIFE IN DEMENTIA CAREGIVERS AFTER USING ONLINE SELF-GUIDED ACCEPTANCE AND COMMITMENT THERAPY

**DOI:** 10.1093/geroni/igae098.2420

**Published:** 2024-12-31

**Authors:** Josey Batura, Jacob Gossner, Ty Aller, Elizabeth Fauth

**Affiliations:** Utah State University, Providence, Utah, United States; Utah State University, Logan, Utah, United States; Utah State University, Logan, Utah, United States; Utah State University, Logan, Utah, United States

## Abstract

**Background:**

At home family care for individuals with dementia averages 24.4 hours per week, with one in four caregivers providing 40+ hours. Caregivers identify positive experiences but also report burnout. Acceptance and Commitment Therapy (ACT) is an empirically validated transdiagnostic approach that aims to increase individuals’ psychological flexibility, or their ability to respond effectively to their internal experiences in the present moment. This study examines the efficacy of a 6-session online, self-guided ACT for Caregivers program. Research Design and

**methods:**

A randomized waitlist-controlled trial included 114 dementia caregivers of whom 28 participated in two semi-structured interviews: at end of sessions (post-test) and 6 weeks later (follow-up; each 45-60 minutes). Participants were recruited from the community. Zoom interviews were transcribed and coded by three researchers using deductive qualitative analysis. Current analyses focused on the self-reported impact of the program on participants’ quality of life. The qualitative subsample included 20 (71.4%) spousal and 8 (28.6%) adult offspring caregivers (Age M= 64.2; SD= 13.3).

**Results:**

Longitudinal coding of post-intervention and follow-up interviews indicated themes of positivity, greater self- compassion, feeling less stressed and calmer, living more vibrantly, and leaning into social supports. They also reported engaging in improved health behaviors.

**Discussion:**

Quality of life is an ideal outcome to examine qualitatively, as ACT aims to enhance QoL, rather than merely reduce negative symptoms. Participation in an online, self-guided ACT program yielded initial and sustained improvements in multiple domains of QoL, and participants emphasized different aspects of improvements in QoL at post-test and follow-up.

